# Transcription of human endogenous retroviruses in human brain by RNA-seq analysis

**DOI:** 10.1371/journal.pone.0207353

**Published:** 2019-01-03

**Authors:** Fang Li, Sarven Sabunciyan, Robert H. Yolken, Doheon Lee, Sanghyeon Kim, Håkan Karlsson

**Affiliations:** 1 The Center for Heart Development, Key Lab of MOE for Development Biology and Protein Chemistry, College of Life Sciences, Hunan Normal University, Changsha, Hunan, China; 2 Department of Neuroscience, Karolinska Institutet, Stockholm, Sweden; 3 Stanley Division of Developmental Neurovirology, Department of Pediatrics, Johns Hopkins University School of Medicine, Baltimore, MD, United States of America; 4 Department of Bio and Brain Engineering, KAIST, Daejeon, Korea; 5 Stanley Medical Research Institute, Johns Hopkins University School of Medicine, Baltimore, MD, United States of America; Charite Universitatsmedizin Berlin, GERMANY

## Abstract

**Background:**

Human endogenous retroviruses (HERV) comprise 8% of the human genome and can be classified into at least 31 families. Increased levels of transcripts from the W and H families of HERV have been observed in association with human diseases, such as multiple sclerosis and schizophrenia. Although HERV transcripts have been detected in many tissues and cell-types based on microarray and PCR studies, the extent of HERV expression in different cell-types and diseases state has been less comprehensively studied.

**Results:**

We examined overall transcription of HERV, and particularly of HERV-W and HERV-H elements in human postmortem brain samples obtained from individuals with psychiatric diagnoses (n = 111) and healthy controls (n = 51) by analyzing publicly available RNA sequencing datasets. Sequence reads were aligned to prototypical sequences representing HERV, downloaded from Repbase. We reported a consistent expression (0.1~0.2% of mappable reads) of different HERV families across three regions of human brains. Spearman correlations revealed highly correlated expression levels between three brain regionsacross 475 consensus sequences. By mapping sequences that aligned to the consensus sequences of HERV-W and HERV-H families to individual loci on chromosome 7, more than 60 loci from each family were identified, part of which are being transcribed. The *ERVWE1*, locus located at chr7q21.2, exhibited high levels of transcription across the three datasets. Notably, we demonstrated a trend of increased expression of overall HERV, as well as HERV-W family in samples from both schizophrenia and bipolar disorder patients.

**Conclusions:**

The current analyses indicate that RNA sequencing is a useful approach for investigating global expression of repetitive elements, such as HERV, in the human genome. HERV-W/H with the tendency of transcription up-regulation in patients suggests potential implication of HERV-W/H in psychiatric diseases.

## Introduction

Whereas approximately 2% of the human genome encodes proteins, 45% consists of transposable elements. These elements are by nature repetitive and can be divided into four different classes, one of which is long terminal repeat (LTR) elements including human endogenous retroviruses (HERV) [1,2]. Based on sequence similarities, at least 31 families of HERV have been identified making up approximately 8% of the human genome [3]. With few exceptions, the functional roles of these repetitive elements remain elusive as the vast majority has no protein coding ability. Transcription of HERV elements have been reported to be both regulated and tissue-specific [4–6]. Our previous analyses of individual loci in the HERV-W family have indicated that some of the transcription is due to transcriptional leakage or to detection of unprocessed pre-mRNA sequences but also due to specific transcriptional initiation and/or terminations within the different repeats themselves [7]. HERV repetitive elements are notoriously difficult to study since they cause ambiguities in genome assemblies, as well as during mapping of transcribed regions. Hybridization-based approaches can be used to detect such transcripts [8–10], but are generally not able to differentiate between specific loci due to extensive cross-reaction of transcripts from different, but highly similar, loci. Combination of microarray and probesets was helpful for minimizing cross-hybridization risks, while probesets were important for profiles display of HERV expression [6]. PCR-based methods have been developed that allow either broad detection (and quantitation) of entire classes of repeats [11–13] or more detailed information of transcripts within single family of repeats based on melting temperature differences detectable during *post*-amplification dissociation curves [5]. Following amplicons sequencing and alignment of coordinates to consensus sequence were additionally needed to identify individual loci [11,13,14]. PCR-assays specific for individual loci have also been successfully employed[7,15], but are, in light of the large number of members of many repeat families, not practical for global analysis. For these reasons, a comprehensive understanding of the extent of transcription in repetitive regions is less clear.

Intriguingly, expression of different subfamilies of HERV has been associated to a range of human diseases. For example, HERV-K/-E transcripts and proteins are expressed at higher levels in a series of cancers[4,16–20]. Transcripts from the HERV-H and HERV-W families have been observed in brain or CSF from patients with multiple sclerosis (MS) [21–23]. We and others have previously reported that transcripts from members of the HERV-W family were expressed in brain, CSF and blood of patients with schizophrenia at a higher level than observed in samples from control individuals [24–27].

Next generation sequencing holds the promise to improve our understanding of the extent of transcription of genomes in a range of different cell-types and disease states. This method entails generating hundreds of millions of relatively short sequence reads (50–150 bases) from various sources of RNA. While the limited read-length can make mapping reads to unique positions a challenge, the method(s) have proven useful for detecting repeat transcript abundances in human diseases. A previous study using whole transcriptome sequencing reported that ~8% of mappable reads originated from repeat sequences in human cortex. In particular, abundant expression of HERV-W elements across human tissues including brain was confirmed by Northern blot [28]. Accumulating evidence suggested applicability of high throughput sequencing in capturing expression of specific HERV families in tissues under pathological conditions, such as cancer [16,29] and MS [30]. Recent studies using such technique unveiled expression profiling of repetitive elements in brain samples of subjects with psychiatric diseases[31,32]. The proportion of transcripts generated from HERV repeat sequence in these samples is however not known.

In the present study we use publicly available RNA-sequencing datasets generated from three regions of human postmortem brain; anterior cingulate cortex, hippocampus and orbitofrontal cortex to investigate transcription of HERV overall and particularlyof the HERV-W and HERV-H families.

## Materials and methods

### Samples

Anterior cingulate cortex, hippocampus and orbitofrontal cortex tissues (Table 1) were dissected from frozen postmortem brains as part of the Stanley Brain Collection, Bethesda, MD, USA. Demographic and clinical characteristics of patients with psychiatric disorder diagnoses and healthy controls were provided in previous studies [28,31,32].

**Table 1:** Reads of sequencing data from three human brain regions.

	Anterior Cingulate	Hippocampus	Orbitofrontal		
Total number	82	58	22		
Sequence mapping	Reads sum (normalized mean)			Consensus sequence length	Genomic size
Human Genome	303,291,401	2,595,667,706	2,773,232,009		
HERV	631,873	4,741,558	3,839,534		
HERV17	4812 (15.96)	39,698 (15.43)	26,626 (9.65)	8626 bp	872,510 bp
LTR17	2540 (8.20)	26,089 (10.20)	15,677 (5.69)	780 bp	482,275 bp
HERVH	52281 (172.23)	292,011 (112.33)	191,257 (120.26)	780 bp	5,593,542 bp

### Library generation and sequencing

Procedures for RNA isolation, library preparation and sequencing have been previously described [28,31,32].

### Sequence data analysis

The sequencing data can be requested at http://sncid.stanleyresearch.org/. 475 individual sequences of representing LTR repeats (consensus sequences of large families and example sequences of small families) were downloaded from Repbase at http://www.girinst.org/repbase and imported into CLC Genomics Workbench 5. This software were subsequently used for alignment of sequence reads to the consensus sequence allowing only unique matches using the following settings; similarity: 0.9, length fraction: 0.9, insertion cost: 3, deletion cost: 3, mismatch cost: 2. Reads were also aligned to the human genome (Hg19) using the same settings but including reads with multiple hits (randomly assigned). To identify transcribed loci, reads matching HERV-W (HERV17-int, LTR17) and HERVH consensus sequences were collected and mapped to the human genome allowing only unique matches.

### Statistical analysis

The nonparametric Mann-Whitney test was used to compare groups using GraphPad Prism (version 3.02) software. A *P* value of < 0.05 was considered significant. Adjustment for multiple testing was not performed in the present study.

## Results

### Global analyses of RNA sequence data

**Fig 1:**
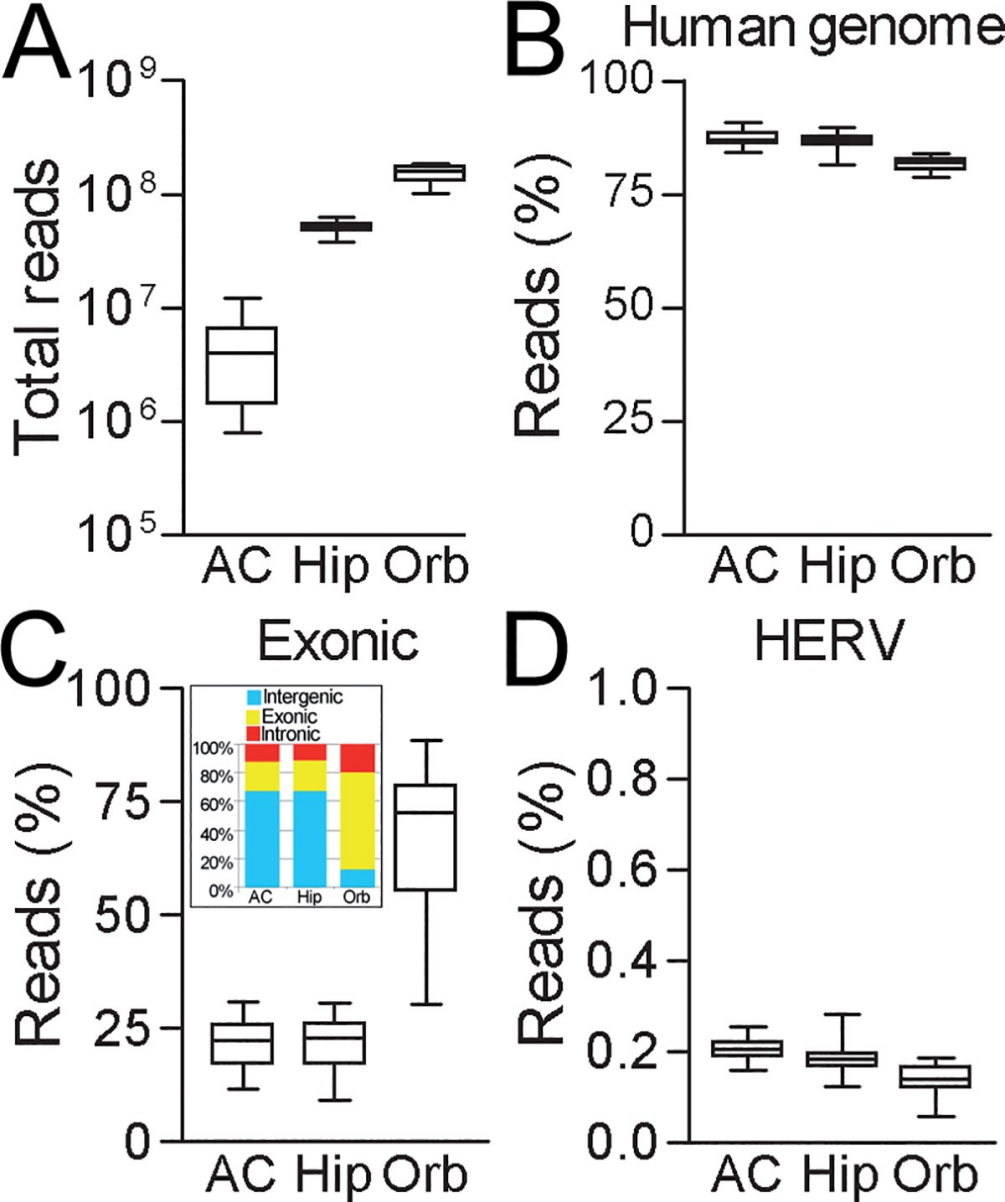
Sequencing reads in brain samples. (A) Total reads obtained from different brain tissues by sequencing. Percentage of reads from different brain tissues aligning to (B) human genome, (C) exonic regions and (D) HERV consensus sequences. Insert of (C) indicated average proportion of reads aligning to intergenic, exonic and intronic regions of genome. AC, anterior cingulate; Hip, hippocampus; Orb, orbitofrontal.

As can be seen in Fig 1A, an average of 4.2 million reads were obtained from each anterior cingulate cortex sample (n = 82). An average of 45 million reads and 126 million reads were obtained from hippocampus (n = 58) and orbitofrontal cortex (n = 22) samples, respectively. Of these reads generated from three different brain regions, 80~90% could be aligned to the human genome (Hg19) allowing for multiple hits (Fig 1B, Table 1). Among reads mapping to a unique position, the proportions of reads aligning to exonic regions of the genome varied considerably between the different datasets. Anterior cingulate cortex and hippocampus samples had an average approximately 22% of reads aligning to exon regions whereas orbitofrontal cortex samples had approximately 70% of reads aligning to exon regions (Fig 1C). Orbitofrontal cortex libraries also contained the lowest proportions of intergenic reads (13%, Fig 1C insert). Interestingly, the average proportions of sequence reads aligning to the different HERV consensus sequences ranged between 0.1~0.2% in all three regions investigated (Fig 1D, Table 1).

### HERV expression in brain regions

**Fig 2:**
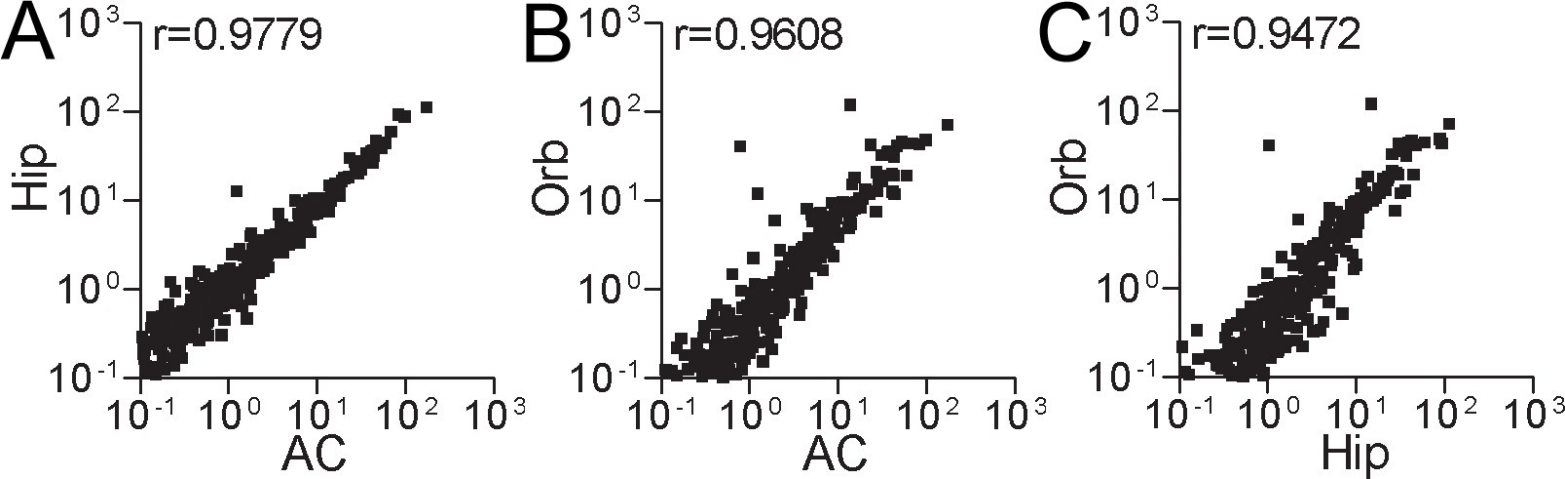
Spearman correlation analyses regarding sequencing reads from different brain samples aligning to 475 repeat consensus sequences. AC, anterior cingulate; Hip, hippocampus; Orb, orbitofrontal.

Normalized expression levels (i.e. number of unique reads mapping to a specific consensus/total number of mappable reads in that sample*10^6^) for each of the 475 HERV sequences were calculated for all samples in each of the datasets. We subsequently calculated average normalized expression levels for each of the three brain regions. Fig 2 displayed pair-wise correlations of these average levels between the three regions. As can be seen from these a varied, over at least three orders of magnitude, expression of the different consensus sequences was detected in all regions with pair-wise correlation coefficients (Spearman rho’s) ranging between 0.9472–0.9779. HERVH (the internal portion of HERV-H) was consistently among the most abundantly expressed HERV members in anterior cingulate cortex and hippocampus samples (Table 1). In the orbitofrontal cortex libraries, however, HERVIP10FH, ranked ahead of HERVH in terms of expression level (data not shown).

### Distribution of HERV throughout the human genome

**Fig 3:**
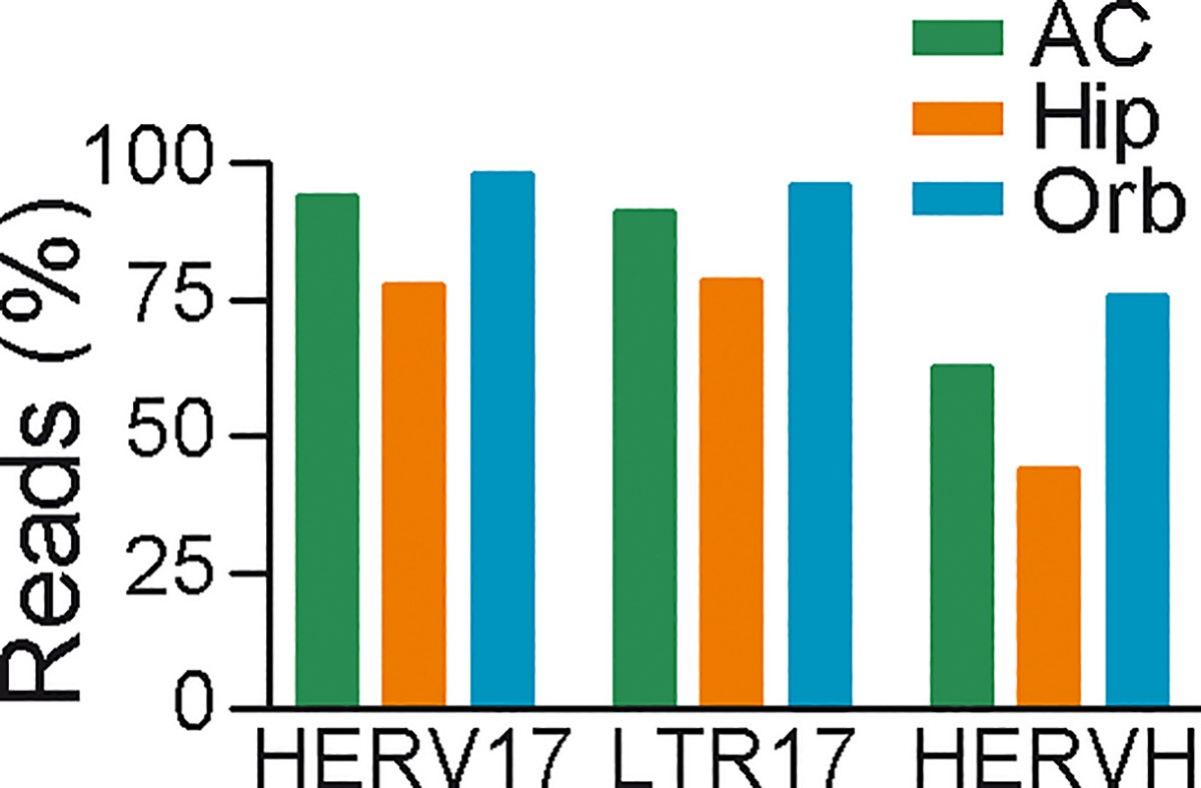
Mapping of repeat families to human genome. Proportion of repeat-reads aligning to the consensus sequences of HERV17, LTR17 and HERVH from different brain samples that could be mapped uniquely to the human genome was indicated. AC, anterior cingulate; Hip, hippocampus; Orb, orbitofrontal.

To examine transcription from individual loci in more detail, we extracted the sequences aligning to HERV17, LTR17 and HERVH (Table 1). These reads were subsequently aligned to the human genome, counting only reads that could be aligned to a unique position. Fig 3 illustrated the proportions of reads from these different repeat families that could be mapped uniquely to the human genome in the three different datasets. There was a considerable variation across both libraries and repeat families. Orbitofrontal cortex had the single-end reads (100 bp) and allowed the largest proportion of reads to be mapped to a unique position followed by anterior cingulate cortex with 76 bp trimmed reads and hippocampus with 50 bp paired-end reads. For reads mapping to HERV17 and the corresponding LTR17, 75–98% could be mapped to unique positions. For reads mapping to HERVH, a smaller proportion (44–75%) could be mapped to a unique position in build 19 of the human genome.

**Fig 4:**
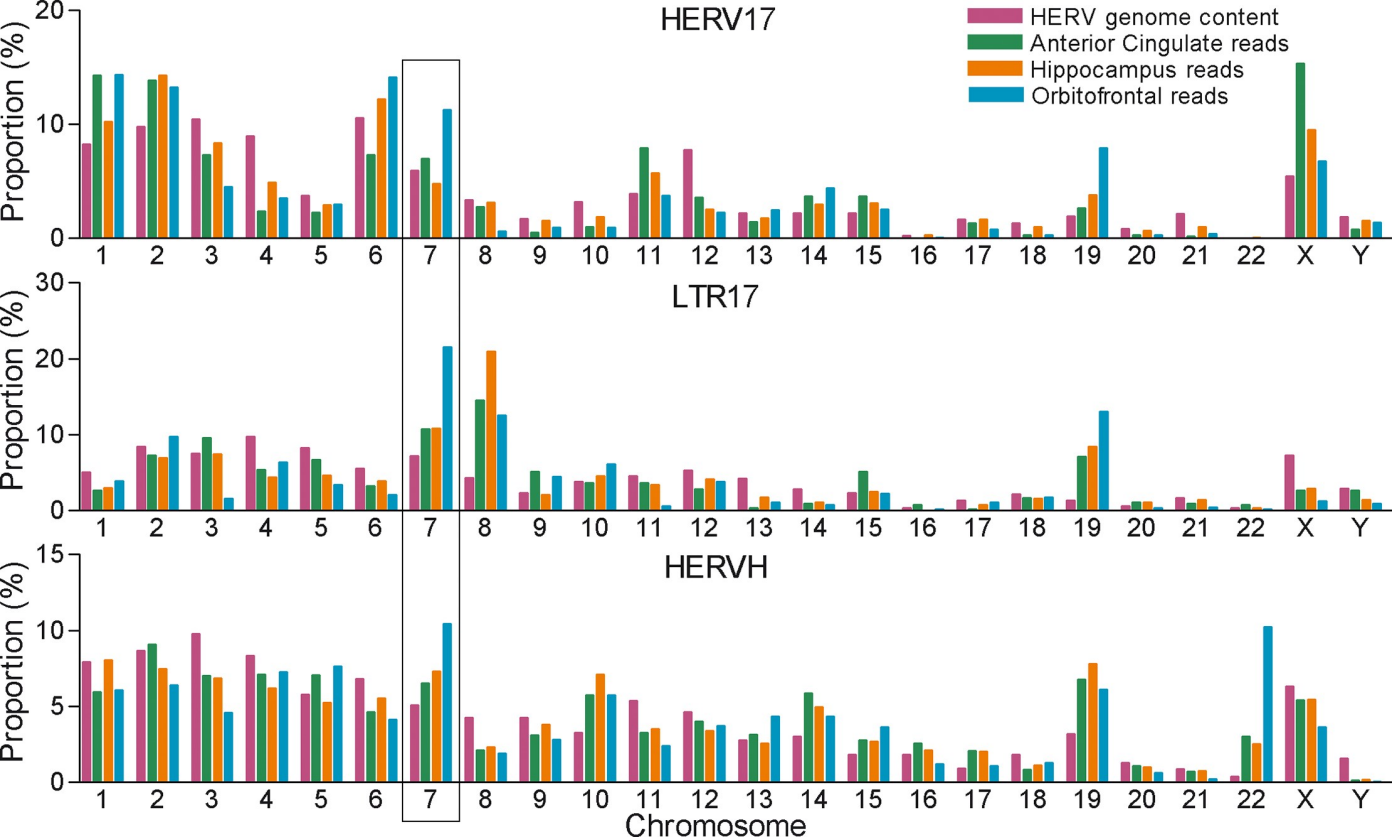
Distribution of HERV17, LTR17 and HERVH repeat sequences and of corresponding normalized reads from anterior cingulate, hippocampus and orbitofrontal samples mapped to individual chromosomes.

We next investigated the distribution of reads mapping to these repeat families across human chromosomes. Proportions of the total number of bases (total genome content) annotated as HERV17-int, LTR17 or HERVH across the human chromosomes were calculated and are indicated in Fig 4 along with proportions of uniquely mapped reads in the different regions. Each of the three HERV family members was present on all chromosomes but with substantial variation. Over 50% of repeated sequences from each subfamily located on chromosomes 1~7, suggesting an unbiased integrations with regard to the chromosome length. The proportion of reads which uniquely mapped to each chromosome was, however, not consistent with the proportion of integrated repeat sequences. For example, ~10% of HERV17 elements inserted on either chromosome 1 or 2, whereas ~15% of HERV17 reads from each of three regions mapped to loci on these chromosomes. In contrast, nearly equal percentages of HERV17 elements (~11%) inserted on either chromosome 3 or 4, but only 2~8% reads across regions mapped uniquely to these loci. Similar observations were also made for LTR17 and HERVH. Notably, large proportions of reads, particularly in orbitofrontal cortex libraries, mapped to chromosome 7. Taken together, these mapping results indicated that not all of the individual loci are transcribed (i.e. have reads mapping to them), but that there is a proportion of HERV elements, that appear to be expressed at high levels. For example, chromosome 7 generated a far larger proportion of reads than would be expected based on its content of HERV-W. Similarly chromosome 22 had very little (0.4%) of the total genome content of HERV-H but generated approximately 10% of the expressed reads that could be mapped uniquely.

### Comparisons of transcription from individual loci

**Fig 5:**
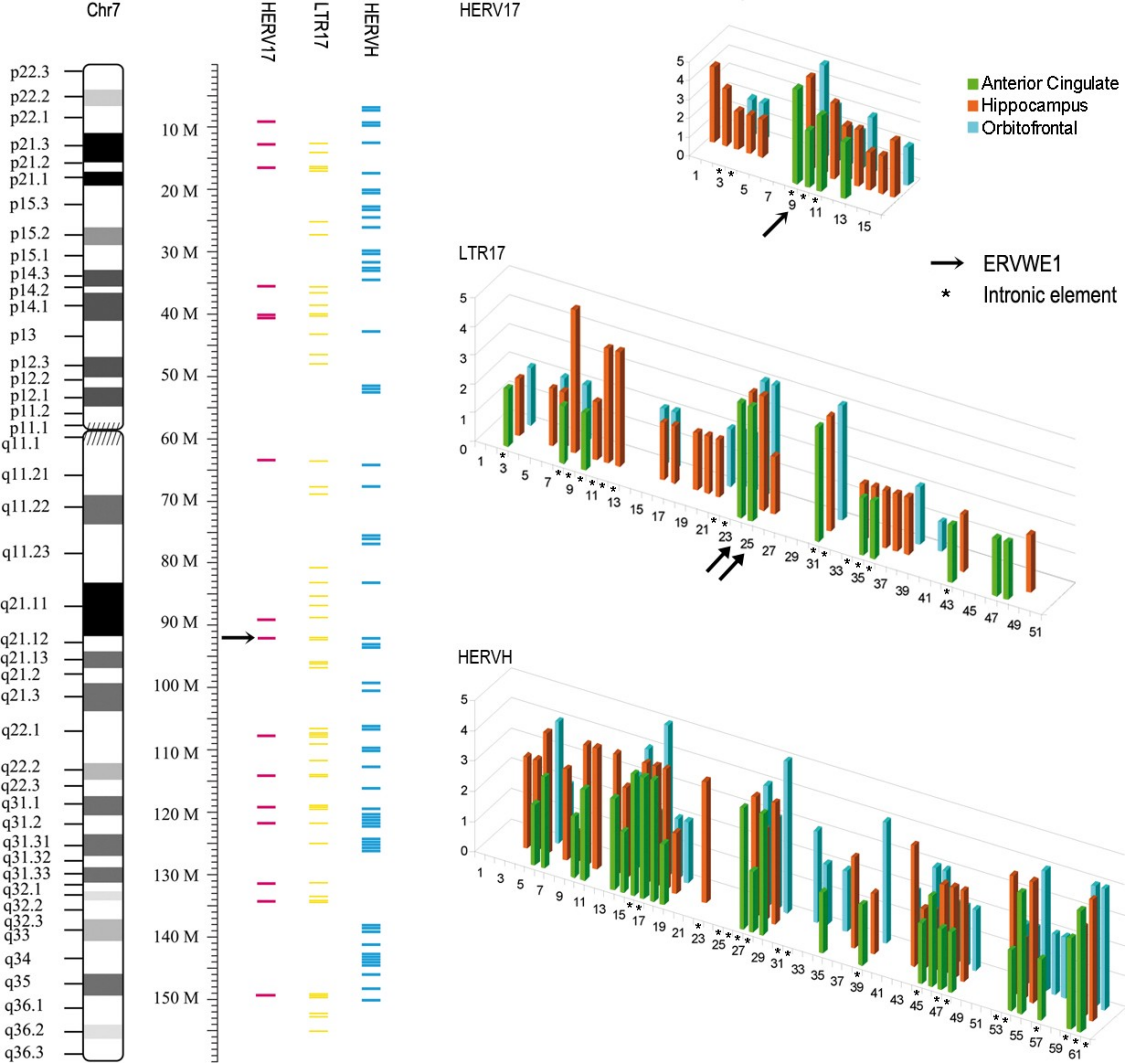
Relative transcript levels of repeat elements in brain samples on chromosome 7. Left, positions of individual HERV17-int, LTR17 and HERVH loci on chromosome 7. Right, relative transcription of each locus from anterior cingulate, hippocampus and orbitofrontal is indicated by the height of the bars. Individual loci located in introns of annotated genes are indicated by an asterisk (*) to distinguish them from loci located in intergenic regions. The *ERVWE1* locus is highlighted by arrow.

In light of these findings we further investigated expression of individual loci representing HERV17, LTR17 and HERVH integrations on chromosome 7. We illustrated loci that exhibited signs of being transcribed in the three different regions, see Fig 5. The relative level of transcription of each locus was evaluated based on arbitrary values: 1~5 indicating low to high numbers of reads mapping uniquely to an individual locus (1 indicated detection of at least 3 reads from a dataset). As shown in Fig 5, it was obvious that not all elements in any one subfamily of repeats showed evidence of expression. 3/16 (19%) loci of HERV17 subfamily, 22/51 (44%) of LTR17 subfamily and 23/62 (37%) loci of HERVH subfamily had no reads aligning to them. With regard to the HERV17 subfamily, the largest proportion of expressed loci was detected in hippocampus, 13/16 loci exhibited transcription. The fewest expressed HERV17 loci were detected in anterior cingulate cortex (4/16). It should be noted that in all regions investigated, reads mapped to the *ERVWE1* locus, encoding syncytin-1 that indicated with an arrow, #9 which scored at the highest level of expression. Moreover, reads mapped not only to intronic loci (indicated by * in Fig 5) but also to intergenic HERV17 loci. Of the 51 LTR17 loci on chromosome 7, 23 showed evidence of transcription in hippocampus. Corresponding numbers for anterior cingulate cortex and orbitofrontal cortex were 11 and 13, respectively. Again, it should be noted that both the 3’ and the 5’LTR in the *ERVWE1* locus, indicated by arrows, showed evidence of being transcribed in all regions investigated. In addition to proviral elements such as *ERVWE1* locus, pseudoelements(lack U3 region of 5’LTR or U5 region of 3’LTR) also appeared to be expressed in all regions. Loci #10 and #11 in the HERV17 mapping, represented elements integrated in intronic regions of the genes *NRCAM* and *FOXP2*, respectively, were both expressed at moderate levels. Accordingly, locus # 31 in the LTR17 mapping, represented the 5’LTR (lacking the U3 region) fragment in intronic region of gene *NRCAM*.Similarly, locus #35 represented the 3’LTR (lacking the U5 region) fragment in an intronic region of gene *FOXP2*. These two LTRs were in correspondence with the HERV17 mapping loci #10 and #11, and exhibited intermediate to high levels of transcription.

Of the 62 loci in the HERVH subfamily on chromosome 7, 24, 30, and 30 showed evidence of transcription in anterior cingulate cortex, hippocampus and orbitofrontal cortex, respectively. A large number of reads mapped to intergenic loci in all regions. It should also be noted that HERVH element #29, located only 2000 bases upstream (in tandem) of the *ERVWE1* locus on the negative strand of chromosome 7 exhibited evidence of being transcribed in all regions investigated.

#### HERV transcription in neuropsychiatric diseases

**Fig 6:**
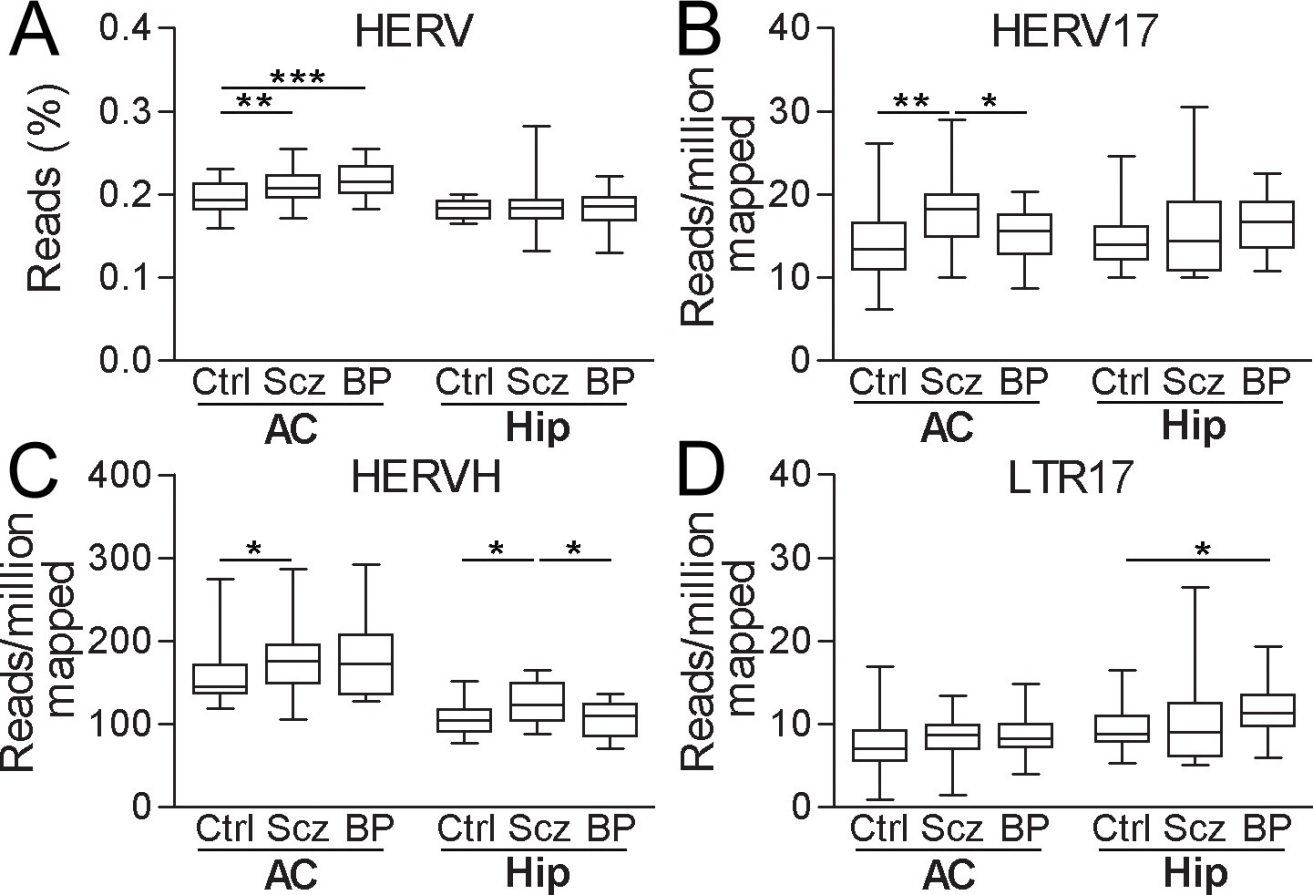
Sequencing reads in brain samples from individuals with psychiatric disorders and control individuals aligning to repeat consensus sequences. Proportion of reads aligning to (A) HERV consensus sequences, normalized reads aligning to (B) HERV17, (C) HERVH and (D) LTR17 consensus sequences in tissues of control and patients. Ctrl, healthy control; Scz, schizophrenia; BP, bipolar disorder; AC, anterior cingulate; Hip, hippocampus. *, p<0.05; **, p<0.01; ***, p<0.001.

Since the anterior cingulate cortex, hippocampus and orbitofrontal cortex samples were obtained from individuals with psychiatric diagnoses and control individuals (Table 2), we finally compared HERV expression across these different diagnostic groups available for each region. To this end, we investigated the normalized read-counts for the total number of HERV sequences in the three different brain regions. Compared to control individuals, larger proportions of HERV reads were observed in anterior cingulate cortex but not hippocampus from schizophrenia (P = 0.007) and bipolar disorder (P<0.001) patients (Fig 6A). In anterior cingulate cortex samples, a larger proportion of normalized reads aligned to subfamily HERV17 (HERV-W) in samples from individuals with schizophrenia as compared to samples from individuals with bipolar disorders (P = 0.024) or healthy controls (P = 0.002). Such differences were not detected in hippocampus (Fig 6B). LTR17, in contrast, exhibited higher normalized read counts in patients with bipolar disorders than from control individuals (P = 0.034) in hippocampus (Fig 6C). Interestingly, HERVH, the most abundantly expressed HERV family, presented with increased normalized read counts in anterior cingulate cortex and hippocampus from patients with schizophrenia as compared to corresponding regions from healthy controls (anterior cingulate cortex: P = 0.027, hippocampus: P = 0.031). Furthermore, normalized read counts of HERVH in hippocampus samples from schizophrenia patients were also higher than those from bipolar disorders patients (P = 0.037) (Fig 6C). However, neither total HERV expression, nor expression levels of the investigated subfamilies differed in orbitofrontal cortex between patients with bipolar disorder and control individuals (data not shown).

**Table 2:** Psychiatric diagnostic groups from three human brain regions.

	Anterior Cingulate	Hippocampus	Orbitofrontal
Diagnosis	Number		
Healthy control	26	15	10
Schizophrenia	32	14	/
Bipolar disorder	24	14	12
Depression	/	15	/
(Total)	(82)	(58)	(22)

## Discussion

With the effectivity of RNA sequencing for characterizing the entire transcriptome in any sample, increasing numbers of studies have recently used the approach to gain insights into the transcription of repetitive regions of human genome[4,28–32]. We here report a consistent expression of different HERV families in publicly available datasets representing three regions of human brain from multiple individuals. Comparisons across the diagnostic groups, schizophrenia, bipolar disorder and controls suggested increased transcription of HERV in both schizophrenia and bipolar disorder. Following mapping of the individual samples to the annotated human genome, we observed major differences between the brain regions investigated. While comparable proportions of reads in the individual datasets were mappable (80–90%) to the human genome, the proportions of reads mapping to exons were considerably higher in the libraries generated from orbitofrontal cortex (75%) than in the anterior cingulate cortex and hippocampus libraries (25%). These differences could be most likely explained by differences in RNA and/or library preparation and not by regional expression differences. Orbitofrontal cortex libraries were prepared from high poly-A enriched total RNA samples that were DNase treated and consequently resulted in the lowest proportions of intergenic reads (13%). Therefore, comparison studies across brain regions using these datasets need careful interpretation of results. These observations also suggested that the orbitofrontal cortex dataset was highly enriched in mature mRNAs with little contaminating genomic DNA. The proportions of reads mapping to the 475 sequences representing known HERV elements in the three datasets also differed, with orbitofrontal cortex samples containing the lowest proportion (0.1%) and the other two datasets averaging around 0.2%. Since HERV elements collectively have been reported to make up approximately 8% of the human genome, the present observation suggest a general repression of transcription in these regions. This is completely in line with the general notion regarding this class of repetitive elements. However, the fact that a consistent proportion of HERV expression was detected in all orbitofrontal cortex samples supports their expression also in a poly-A enriched fraction of RNA. In light of the differences observed between the orbitofrontal cortex and the hippocampus/anterior cingulate libraries, the highly correlated expression levels observed across the different consensus sequences was somewhat surprising. It should however be kept in mind that these correlations were based on very large numbers of reads obtained from large numbers of individuals and thus represented gross averages across consensus sequences that might hide individual differences in terms of transcribed loci within the different families.

Data from our attempt to map reads aligning with three different consensus sequences representing the HERV-W (HERV17-int, LTR17) and the HERV-H (HERVH) families illustrated the difficulties associated with studies on repetitive elements. The HERV-H family has approximately 6 times more members than the W family, see the genomic size in Table 1. Many highly similar members, lead to a smaller proportion of uniquely mapped reads. Mapping of expressed sequences in the HERV-W and HERV-H families indicated that transcription was not merely a consequence of genome content but rather implied that some elements are expressed whereas others are more or less quiescent. This observation was in agreement with our previous studies using PCR directed at specific HERV-W loci [7]. Moreover, our attempts to map transcription to individual loci along chromosome 7 further supported this notion. It was evident that several loci are expressed, not only in cingulate and hippocampus but also in the orbitofrontal cortex samples. These were not solely restricted to intronic loci but included several intergenic loci in both HERV-H and -W families. Moreover, we detected expression of both proviral elements with intact LTRs and psudoelements lacking the regulatory U3 region in their 5’LTRs. This observation thus provides independent verification of our previous finding using locus-specific PCR [7]. Of particular interest in terms of expression was obviously the *ERVWE1* locus in the HERV-W family. The *env* gene in this locus encodes the fusogenic protein; syncytin-1 [33], normally expressed at high levels in the syncytiotrophoblast layer of the human placenta [34–36]. Interestingly, a number of independent studies have suggested that this protein is aberrantly expressed in multiple sclerosis brains in areas of ongoing demyelination [23,37,38]. Our mapping efforts clearly indicated that this locus had some degree of expression in all three libraries investigated.

In light of previous reports by us and others of increased expression of HERV in schizophrenia, it was interesting to note that the RNA sequencing data on postmortem indicated a slightly larger proportion of reads mapping to HERV in general in bipolar disorder. Increased levels of transcripts from HERV-W in schizophrenia thus provides verification of previous reports in postmortem brain tissues obtained by PCR [26] and extended these to include also bipolar disorder. Not only HERV-W but also HERV-H was expressed at increased levels in schizophrenia suggesting that other families were differentially expressed in schizophrenia. The majority of these differences between diagnostic groups were observed in the anterior cingulate cortex libraries. While these represented the largest number of individuals they also contained the smallest number of reads per sample. Disease specific differences for both HERV-W (LTR17) and HERV-H families were however observed also in hippocampus libraries which contained far more sequence reads per sample but represented fewer individuals. It should be noted that corrections for multiple testing were not performed here. Considering the four comparisons presented in Fig 6, significances indicated by *, p<0.05, might not survive correction. Apparently a trend for elevated expression of overall HERV or HERV-W/-H subfamilies in samples from patients with psychiatric disease was in line with previous findings [24–27]. No disease-related differences in terms of HERV expression was detected in the orbitofrontal cortex libraries. These included the smallest number of individuals but by far the largest number of reads per sample. Also the orbitofrontal cortex libraries appeared to contain a representation of sequences that differed from the cingulate and hippocampus libraries in that exons a far more represented. It is not clear if the lack of disease-related differences in the orbitofrontal cortex samples is caused by a lack of power or if these differences are detectable only in non-polyadenylated RNA, thus, more sample studies particularly by RNA-seq method are required to replicate the analysis.

## Supporting information

S1 Dataset Data set supporting information file of 475 repeat sequences. (XLS)
